# The Outcome of Surgical Intervention (Ventriculoperitoneal Shunt and Endoscopic Third Ventriculostomy) in Patients With Hydrocephalus Secondary to Tuberculous Meningitis: A Systematic Review

**DOI:** 10.7759/cureus.25317

**Published:** 2022-05-25

**Authors:** Roopa Chalasani, Mastiyage R Goonathilake, Sara Waqar, Sheeba George, Wilford Jean-Baptiste, Amina Yusuf Ali, Bithaiah Inyang, Feeba Sam Koshy, Kitty George, Prakar Poudel, Lubna Mohammed

**Affiliations:** 1 Research, California Institute of Behavioral Neurosciences and Psychology, Fairfield, USA; 2 Pediatrics/Internal Medicine, California Institute of Behavioral Neurosciences and Psychology, Fairfield, USA; 3 Pediatrics, California Institute of Behavioral Neurosciences and Psychology, Fairfield, USA; 4 Internal Medicine, Chitwan Medical College of Medical Science, Chitwan, NPL; 5 Internal Medicine, California Institute of Behavioral Neurosciences and Psychology, Fairfield, USA

**Keywords:** tuberculous meningitis, tb meningitis, hydrocephalus, ventriculoperitoneal shunt, endoscopic third ventriculostomy

## Abstract

The objective of this study is to analyze the outcome of the safety and efficiency of the surgical interventions (ventriculoperitoneal shunt [VPS] and endoscopic third ventriculostomy [ETV]) in patients with hydrocephalus due to tuberculous (TB) meningitis. A systematic literature search has been conducted using PubMed, Google Scholar, PMC, and ScienceDirect databases from 2001 to 2022 April. A total of 16 studies have been included, irrespective of their design. These studies include patients diagnosed with hydrocephalus secondary to TB meningitis (TBM) treated with VPS or ETV. A systematic review was conducted to determine the efficiency of surgical procedures based on the outcomes and complications associated with these procedures. A total of 2207 patients (aged one month to 68 years) have been included in this study, out of which 1723 underwent VPS and 484 underwent ETV. The overall success rate in the VPS group varied from 21.1% to 77.5%. The overall success rate in the ETV group ranged from 41.1% to 77%. The overall complications rate in the VPS group varied from 10% to 43.8%, and the complications rate in the ETV group varied from 3.8% to 22.5%. After ruling out the significant differences in the average percentages of outcomes and complications followed by VPS and ETV, ETV is suggested in patients with chronic phases of illness because the chances of ETV failure are high during the initial stage. The uncertainty of the ETV gradually decreases over time. To attain favourable long-term outcomes with ETV in patients with TBM hydrocephalus (TBMH), ETV should be performed after chemotherapy, anti-tubercular treatment, and steroids. In addition, ETV is considered beneficial over VP shunt as associated long-term complications are significantly less compared to VP shunt. In contrast, VP shunt is suggested as a modified Vellore grading which shows a more favourable outcome in patients with acute illness than ETV.

## Introduction and background

Tuberculous meningitis (TBM) is a bacterial infection of the central nervous system involving the meninges of the brain and spinal cord. Mycobacterium tuberculosis is the causative organism of TBM. Hydrocephalus is the most common complication of TB meningitis, affecting children more than adults [1]. It is almost always present in patients who have had the disease for four to six weeks and occurs at an early stage of the disease process [1]. The hydrocephalus in patients with tuberculous meningitis could be either the communicating type or the obstructing type, the former being the more common [2]. The developmental issue of the obstructive type of hydrocephalus in tuberculous meningitis is either due to blockage of the fourth ventricle by thick exudates or leptomeningeal scarring [3]. The early stage of this communicating type of hydrocephalus causes thick gelatinous exudates to block the subarachnoid spaces in the base of the brain (more significant in the interpeduncular and ambient cistern). The later stage of the communicating type of hydrocephalus causes the exudates, which leads to dense scarring of the subarachnoid spaces. Communicating hydrocephalus may also result from an overproduction of CSF or secondary to reduced absorption of CSF. Communicating hydrocephalus is seen more recurrently in patients with TBM [3]. According to body weight, the medical management of TBM hydrocephalus (TBMH; communicating type) includes ATT (standard four-drug antitubercular therapy consisting of rifampicin, ethambutol, isoniazid, and pyrazinamide), along with steroids (dexamethasone given if CT showed thick basal exudates and there was evidence of infarcts) [2], and dehydrating agents acetazolamide, furosemide, and mannitol [1]. The surgical management of TBMH includes endoscopic third ventriculostomy (ETV) and ventricular shunting (VA, VP, VPL, LP), most commonly ventriculoperitoneal (VP) shunting, which has been the procedure of choice so far [4]. Attempts to relieve pressure symptoms in infants with enlarged heads and adults with papilloedema and high lumbar cerebrospinal fluid (CSF) include cerebellar decompression, lateral and third ventriculostomy, and short-circuits between the ventricular system and subarachnoid space of the cerebral hemispheres [5]. However, the best plan to relieve the communicating hydrocephalus is to persist with intrathecal and systemic streptomycin [5]. High cerebrospinal fluid protein levels delay shunting.

Nevertheless, ventriculoperitoneal shunt (VPS) surgery complications in patients with TBMH are high, with frequent shunt obstructions and shunt infections requiring repeated revisions [4]. Therefore, the clinical grading system determines the patient's treatment strategy [3]. The most commonly used system is the Vellore grading of TBMH (Table 1), proposed by ​​Palur et al. [6]. Alongside, Table 2 briefly discusses modified Vellore grading of patients with TBMH.

**Table 1:** Vellore grading of tuberculous meningitis hydrocephalus patients.

Grade	Neurological status
Grade 1	Headache, vomiting, fever ± neck stiffness. No neurological deficit. Normal sensorium.
Grade 2	Neurological deficit present, normal sensorium.
Grade 3	Altered sensorium but easily arousable. Dense neurological deficit may or may not be present.
Grade 4	Deeply comatose, decerebrate or decorticate posturing.

**Table 2:** Modified Vellore grading of TBMH. TBMH: tuberculous meningitis hydrocephalus [7].

Grade	Neurological status	GCS SCORE
Grade 1	Headache, vomiting, fever. No neurological deficit	15
Grade 2	Neurological deficit present	15
Grade 3	Neurological deficit may or may not be present	9-14
Grade 4	Neurological deficit may or may not be present	3-8

## Review

Methodology

The Preferred Reporting Items for Systematic Reviews and Meta-Analysis (PRISMA) guidelines 2020 were followed in this systematic review [8], and the population, intervention, comparison, and outcome (PICO) format was included in this study pattern.

The eligibility criteria of the studies in our survey can be found in Table 3.

**Table 3:** The eligibility criteria of the studies included and excluded in our survey. VPS: ventriculoperitoneal shunt, ETV: endoscopic third ventriculostomy.

Inclusion criteria	Exclusion criteria
Articles published in the English language with DOI number.	Non-English publications.
Study age - 2001 January to 2022 April.	Study age - before 2001 studies.
Study population - human infants, children, adolescents, and adults.	Study population - animal study.
Eligible study - patients suffering from TBMH and those who underwent either VPS/ETV.	Ineligible study - patients with alternative diagnosis to tubercular meningitis, i.e., cryptococcal meningitis, pyogenic meningitis.
Study type - cohort studies (prospective and retrospective) randomised control trials, systematic reviews.	Study type - literature reviews, case reports, case series, editorials, incomplete peer reviews.

Information sources, search strategy and data extraction process

A systematic literature search has been conducted using PubMed, Google Scholar, PMC, and ScienceDirect databases using the relevant keywords and MeSH strategy mentioned below (Table 4). A total of 16 studies have been included irrespective of their design and having been diagnosed with tuberculous meningitis and treated with VPS surgery or endoscopic third ventriculostomy (ETV). Two researchers worked independently to identify and extract the data. Quality assessment of each study is conducted using appropriate quality appraisal tools (NOS - Newcastle Ottawa Assessment Scale for Prospective and Retrospective Cohort Studies and Critical appraisal guide for Systematic Reviews (randomised studies) from April 21 to 30, 2022. After removing all the duplicates manually and via Endnote, the author's inclusion and exclusion criteria were used to evaluate the study. All the irrelevant studies have been omitted. The third author resolved the differences of opinion between the first two authors. After a complete analysis, 16 articles have finally been considered in this review.

The purpose of the study is to contemplate the outcome, safety, efficiency of surgeries (VPS and ETV), and complications of patients who underwent either ventriculoperitoneal shunt or endoscopic third ventriculostomy. The efficiency of procedures is based on the resolution of signs and symptoms and also on Vellore grading of patients with TBMH.

The search strategy of different databases using relevant keywords and MeSH strategy is summarised in Table 4.

**Table 4:** Search strategy of different databases.

Databases	Keywords	MeSH strategy	Filters applied
PubMed	Endoscopic third ventriculostomy, ventriculoperitoneal shunt, hydrocephalus, TB meningitis, tuberculous meningitis	Endoscopic third ventriculostomy OR ("Ventriculostomy/therapeutic use"[Majr] OR "Ventriculostomy/therapy"[Majr]) AND Ventriculoperitoneal shunt OR ("Ventriculoperitoneal Shunt/statistics and numerical data"[Majr] OR "Ventriculoperitoneal Shunt/therapeutic use"[Majr] AND ("Hydrocephalus/surgery"[Majr] OR "Hydrocephalus/therapy"[Majr]) AND TB meningitis OR ("Tuberculosis, Meningeal/cerebrospinal fluid"[Majr] OR "Tuberculosis, Meningeal/complications"[Majr] OR "Tuberculosis, Meningeal/drug therapy"[Majr] OR "Tuberculosis, Meningeal/surgery"[Majr] OR "Tuberculosis, Meningeal/therapy"[Majr])	Humans, English, child: birth-18 years, child: 6-12 years, adolescent: 13-18 years, adult: 19+ years, young adult: 19-24 years, middle aged: 45-64 years, Study age - 2001 Jan- 2022 April
Google Scholar	Endoscopic third ventriculostomy, ventriculoperitoneal shunt, hydrocephalus, TB meningitis	"Ventriculoperitoneal shunt" OR "endoscopic third ventriculostomy" AND “tubercular meningitis" AND “hydrocephalus."	NONE
PMC	Not used	"Ventriculoperitoneal shunt" OR "endoscopic third ventriculostomy" AND "TB meningitis" AND "hydrocephalus."	NONE
ScienceDirect	Endoscopic third ventriculostomy, ventriculoperitoneal shunt, hydrocephalus, TB meningitis	"Ventriculoperitoneal shunt" OR "endoscopic third ventriculostomy" AND "TB meningitis" AND "hydrocephalus."	Research articles, open access and open archive

Results

Quality Assessment

Quality assessments of the reviews have been performed based on the guidelines mentioned below. In addition, articles that met at least 70% of the criteria have been included.

We followed the guidelines of the Newcastle Ottawa Assessment Scale for prospective and retrospective cohort studies: (1) Was the exposure and outcome of interest clearly explained? (2) Exposed people? (3) Non-exposed people? (4) The outcome of interest not present at the start of the study (5) Were the people similar? (6) Were the exposure and outcomes measured the same way? (7) Was the follow-up done correctly? (8) Was the follow-up long enough and sufficient enough? (9) Was this study published in an indexed journal? Outcome-based on: YES or NO.

The critical appraisals for systematic review are as follows: (1) Aim of the research; (2) Keyword explanation; (3) MeSH strategy; (4) Did the authors describe all the databases they used to collect the data? (5) Inclusion and exclusion criteria; (6) Did the authors check the quality (critical appraisal) of each study they included in the article? How did they critically appraise it? (7) Is the article published in a reliable database? (8) Were multiple authors involved in data extraction and quality appraisal? (9) Cochrane risk of bias assessment tool. Outcome-based on: YES, PARTIAL YES, NO.

Risk of Bias

The risk of bias in the considered studies has been briefed in Table 5.

**Table 5:** Risk of bias. + = low risk bias, ? = not mentioned.

Study	Detection bias, outcome	Attrition bias, outcome	Reporting bias, complications
Lampre-ht et al. [9]	+	+	+
Husain et al. [10]	+	+	+
Singh et al. [11]	?	+	?
Jha et al. [12]	+	+	?
Figaji et al. [13]	+	+	+
Sil and Chatterjee [14]	+	+	+
Srikantha et al. [15]	?	+	?
Chugh et al. [16]	+	+	?
Yadav et al. [17]	+	+	+
Peng et al. [18]	+	+	+
Savardekar et al. [19]	+	+	+
Goyal et al. [20]	+	?	+
Kankane et al. [21]	+	+	+
Rizvi et al. [22]	+	+	+
Aranha et al. [4]	+	+	+
Bhushan et al. [3]	?	?	+

**Figure 1:**
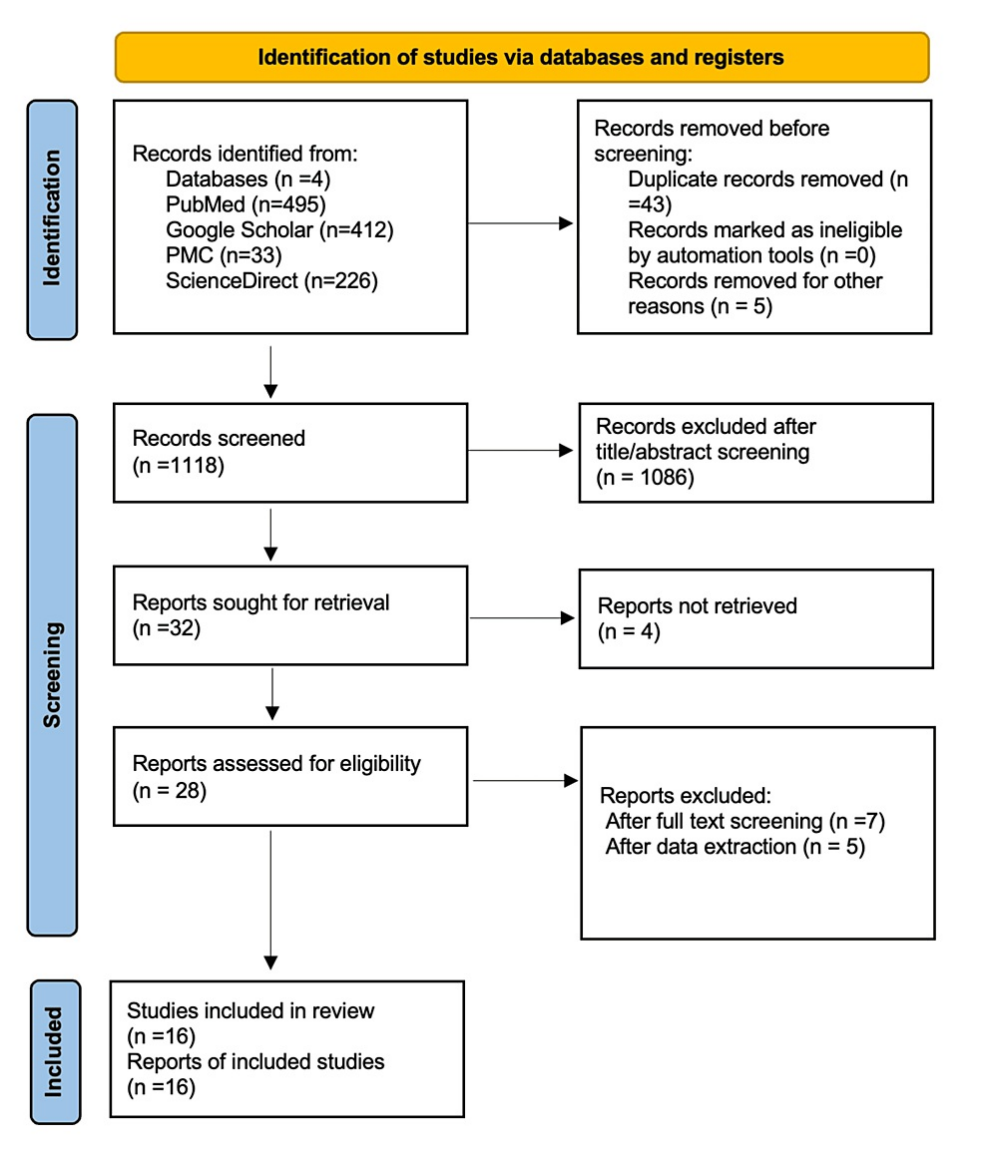
PRISMA flow diagram.

A summary of study selection using PRISMA flow diagram can be found in Figure 1.

Studies of patients with TBMH who underwent either VPS or ETV can be found in Table 6.

**Table 6:** Studies of patients with TBMH who underwent either VPS or ETV. TBMH: tuberculous meningitis hydrocephalus, VPS: ventriculoperitoneal shunt, ETV: endoscopic third ventriculostomy.

S.no	Study	Year of publication	Procedure (VPS/ETV)	Patient details	Outcome	Complication	Comment
1	Lamprecht et al. [9]	2001	VPS	Age – 4 to 131 months. Communicating -27 (41.5%), non-communicating -38 (58.5%). Grade 2 – 29(44.6%), Grade 3 – 36 (55.4%).	Total cases with TBMH – 65. The outcome in shunted TBMH- Good-10(15.4%), moderate disability –26(40%), severe disability – 15(23.1%) vegetative – 6(9.2%) dead – 8 (12.3%). The outcome in the type of hydrocephalus – good outcome and mortality in communicating type – 1 (3.7%) and 5 (18.5%). Good outcomes and mortality in non-communicating type – 9 (23.7%) and 3 (7.9%). The outcome in patients with GCS. 3-5 at the presentation (n=12) Good outcome – 0 Dead – 3 (25%).	Shunted patients -32.3%. Shunt infection – 9 (13.5%), shunt obstruction – 9 (13.5%), over shunting – 2 (3.1%). Wound disruption -1 (1.5%).	VPS has a higher incidence of complications in patients with TBMH rather than in patients with non-tuberculous hydrocephalus shunt surgery. However, they have indicated early VP shunt in patients with non-communicating hydrocephalus.
2	Husain et al. [10]	2005	ETV alone – 19, ETV +monroplasty – 2, ETV + septoplasty – 2, ETV with decompression/biopsy of tuberculoma – 2, ETV with abscess drainage-1.	Age – 5 months – 68 years. 15 males and 13 females.	The total number of TBMH cases – 28. Outcome – Success rate – 68% (19/28), acceptable – 18% (5/28), satisfactory – 50% (14/28), unsatisfactory – 32% (9/28).	Complication rate: 10%. CSF leak: 2 cases responded to intermittent lumbar drainage and oral acetazolamide (20–25 mg/kg per day in divided doses for 2–3 weeks). Perioperative bleed: 1 case-controlled endoscopically followed by EVD for five days, and the patient showed complete recovery over two weeks.	Suggestion-ETV should be regarded as the first surgical option in TBMH as the outcome was satisfactory (50%). Based on the clinical grade, ventriculoperitoneal shunt surgery and EVD should be reserved for patients with ETV failure.
3	Singh et al. [11]	2005	ETV	6 months – 32 years. Grade I – 6 patients, grade II – 7 patients, grade III – 22 patients.	The total number of TBMH patients – 35. The overall success rate of ETV was 77%. Early recovery – 60% of patients. Delayed recovery – 17% of patients. In a clinical recovery – the outcome of success rate in patients with a thin transparent floor of the third ventricle is 87%, whereas it was 74% in patients with a thick or granular floor. There was no significant statistical difference.		They have suggested ETV as an alternative way of managing hydrocephalus and is worth trying before subjecting the patients to VP shunt as they have observed fine results in patients with both obstructed and communicating hydrocephalus.
4	Jha et al. [12]	2007	ETV	Age – 9 months to 40 years. 11 male and 3 female patients.	The total number of patients with TBMH -14. Outcome – The success rate of patients who underwent ETV – 64.2% (9/14) cases.		The presence of advanced grade clinical grade, extra CNS TB, dense adhesions in the prepontine cistern, and unidentifiable third ventricle floor anatomy leads to the failure of ETV.
5	Figaji et al. [13]	2007	ETV/fenestrations/endoscopic biopsy	Age – <12years	The total number of patients with TBMH – 17. Success rate: 41.1% (7/17). Failure rate: 29.4% (5/17). Five patients could not undergo endoscopic third ventriculostomy due to abnormal anatomy. There were five fenestration procedures, three of which were successful. Endoscopic biopsy of two tuberculomas failed to yield a bacteriological result.	Complication rate -11.7% CSF leak was present in 2 cases (In one patient, the CSF leak led to the later development of bacterial meningitis, which was successfully treated). In one patient, brisk venous bleeding was found. This was controlled with irrigation, but visibility was significantly obscured, and the ETV could not be continued safely. After five days, the endoscopic procedure was repeated. Visibility had by then improved, ETV was performed, and the hydrocephalus was successfully treated.	Although ETV is technically possible in this situation, the patients must be adequately selected for the procedure to ensure optimal treatment and that the surgeon has experience with complex cases.
6	Sil and Chatterjee et al. [14]	2008	VPS	Age – 1 month to 12 years. Grade II: 22 (62.5%), Grade III: 12 (37.5%).	Total number of patients with TBMH – 32. Good outcome: 8 (25%) children, moderate disability (cognition and ocular motility disorders): 15 (46.9%) children, severe disability: 5 (15.6%) children, vegetative state: 1 (3.2%) and death: 3 (9.3%) children. Patients in Palur grade II had comparatively better outcomes in each grade.	Shunt infection: 5 (15.6%). Shunt revisions due to blockade: 14 (43.8%) patients.	They recommended that the VP shunt will remain as the only armamentarium in the arsenal of the neurosurgeon for treating this disease even if it gets replaced by a third ventriculostomy in the treatment of other forms of hydrocephalus.
7	Srikantha et al. [15]	2009	EVD ± VPS direct shunt -52 patients. EVD followed by shunt – 43 patients.	Age – 1-55 years	The total number of cases with TBMH – 95. Patients with the favourable short-term outcome: 33% of cases (age older than three years and duration of altered sensorium ≤3 days, GCS score > or equal to 12 at the time of discharge were predictive of favourable short-term outcome). Patients with the favourable long-term outcome: 45% cases (Glasgow Coma Scale score at presentation was predictive of long-term outcome. GCS scores of 7 or 8 at presentation had a favourable follow-up GOS score (4 or 5).		The first management choice for grade 4 patients with hydrocephalus is VP shunt implantation, ATT and steroids. VP shunt should be considered even in patients who do not show improvement with an EVD. NOTE – Presence or absence of infarcts or basal exudates, duration of symptoms and GCS score at presentation did not correlate with short-term outcome. Age, duration of symptoms or altered sensorium, and presence or absence of infarcts or basal exudates did not correlate with long-term outcomes.
8	Chugh et al. [16]	2009	ETV	Age – 7 months to 52 years	The total number of cases with TBMH – 26. The overall success rate was 73.1%. The outcome of ETV was observed to have a statistically significant correlation with the stage of illness and the presence of intraoperative cisternal exudates. A better outcome for ETV was observed in patients on ATT for an extended period preoperatively.		Suggestion-ETV should be considered the first surgical option for cerebrospinal fluid diversion in patients with TBM with hydrocephalus.
9	Yadav et al. [17]	2011	ETV	Age - 6 months – 76 years	The total number of cases with TBMH(Obstructive) – 59 cases. The overall success rate, after ETV alone, was 58% (34 patients). After ETV plus lumboperitoneal shunt: 80% (47 patients).	Total patients with blocked stoma – 3 (5.1%) CSF leak = 6 patients (10.1%) Total patients with associated malnutrition- 31 (53%) Total patients with complex hydrocephalus – 13 (22%)	ETV was safe and effective in TBM hydrocephalus. Significant causes of failure to improve-complex hydrocephalus and associated cerebral infarcts. Good results were observed in better grades. Results of ETV were better in patients without cisternal exudates, good nutritional status, and thin and identifiable floor of the third ventricle compared to cases with cisternal exudates, malnourished, thick unidentifiable floor, respectively, although the difference was statistically insignificant.
10	Peng et al. [18]	2012	VPS ± EDV	1 month – 14 years		Shunt related complications – 6/19 (31.57%), revisions required following shunt block – 3/19 (15.8%). Patients with complications secondary to infections – 2/19 (10.5%) [including erosion of skin (n=1), pneumonia (n=2)] subdural effusion and ventricular haemorrhage – 1/19 patients (5.3%).	Comment based on study demonstration - direct VP shunt placement could improve the outcome in Grade IV TBMH. The response to EVD is not a dependent indication for selecting the patients who would benefit from shunt surgery.
11	Savardekar et al. [19]	2013	VPS	Age – 4 months – 11 years. TBMH Grade III: 21, TBMH Grade IV: 5.	Overall, 26 cases of TBMH. After 3 months: In TBMH Grade III Good outcome: 71.4% (15/21), mortality: 9.5% (2/21). In TBMH Grade IV good outcome: 20% (1/5). Overall good outcome: 61.5%, mortality: 60% (3/5).	Complication rate: 23.5% (6/26). Shunt blockage/malfunction – 2 patients, shunt infection – 2 patients, intraventricular haemorrhage – 2 patients.	Their viewpoint was that direct VP shunt placement is a riskless and successful option in poor-grade patients of TBMH, with a low complication rate.
12	Goyal et al. [20]	2014	VPS and ETV each in 24 cases.	Age – <18 years	The total number of patients with TBMH is 48. The overall success rate in patients who underwent VPS – 13(54.2%), mortality – 2 (One patient – Vellore grade 4, GCS – 6 and another patient – Vellore grade 3 died in the postoperative period due to associated miliary tuberculosis. The overall success rate in patients who underwent ETV – 41.7% (10 cases). In ten cases (41.7%), a VP shunt was done in the post-operative period for ETV failure. Two patients were lost in the follow-up period. Mortality – 2 cases. The first patient (Vellore grade 3) expired due to an associated poor chest condition. In contrast, another patient was discharged in satisfactory condition, later reported to us for CSF leak and died due to fulminant meningitis. ETV failure was more in the young age group (<2 years).	Repositioning of shunt – 16.7% (4 cases). The average complication rate in the ETV group is 16.75%. In the ETV group, CSF leak was noted in seven cases (29.1%). Two patients developed meningitis (8.33%), out of which one patient eventually died. Three patients had a bulge (12.5%) at the ETV site. Shunt-related complications occurred in four (17%) patients and consisted of an obstruction at the lower end of the shunt in three (13%) cases, leading to revision, and one (4%) patient had an infection at the shunt chamber site, leading to skin excoriation and meningitis.	The relative uncertainty of ETV failure is higher than that for shunt, but the uncertainty becomes progressively further down with time. Therefore, if patients pull through the early high-risk period, they could experience long-term survival advantages devoid of lifelong shunt-related complications.
13	Kankane et al. [21]	2016	VPS	Age – 3 months-14 years	Total number of cases with TBMH – 50, with grade 3 and 4. In grade 3 – outcome – 77.5%, mortality – 0%. In grade 4-outcome – 30%, mortality – 10%. Overall outcome – 68%.	The complication rate was 10%	They suggested the direct placement of the VP shunt in Grade 3 and 4 cases with TBMH without intervening in EVD, and the result was good, with a low complication rate.
14	Imran Rizvi et al. [22]	2017	VPS		The total number of cases with TBHM-1038. Overall – 48.4% Good outcome (GOS 5 and 4), following ventriculoperitoneal shunt, was observed in 58.26% of patients, 78.57% of patients in grade 1, 65.35% in grade 2 and 67.9% in grade 3 achieved a good outcome while only 31.51% in grade 4 could achieve a good outcome. On subgroup analysis, 61.08% of HIV-negative patients achieved a good outcome as compared to only 25% of HIV-positive patients. There were 18.03% deaths in the HIV-negative group as compared to 66.67% deaths in the HIV-positive group after shunt surgery.	Complications following VPS were 22.11% shunt blockage, leading to shunting revision, which was the most common complication.	The outcome, following VPS, depends on the clinical severity of TBM. HIV-infected patients have a worse prognosis when compared with HIV uninfected patients. Compared to children, corresponding data is sparse for adult patients with tuberculous meningitis.
15	Aranha et al. [4]	2018	VPS or ETV each in 26 cases	Age – <18years	Fifty-two paediatric patients with TBMH. The success rate in the ETV group was 65.4% (17/26), and in the VP shunt group: 61.54% (16/26). The failure rate in the ETV group was 34.6% (9/26), and in the VP shunt group: 38.4% (10/26). Two cases of mortality were observed in each group.	In the ETV group, one case had a CSF leak which was resolved on conservative management with lumbar drainage. In the VPS group, shunt-lower end malfunction – 6, ventricular end malfunction – 1, shunt tract infection – 3.	They found comparable ETV results in communicating hydrocephalus and obstructive hydrocephalus. In addition, they suggested that it can be performed effectively in communicating hydrocephalus, high CSF cell counts, and protein levels, despite an indistinct third ventricular floor anatomy. So, ETV should be attempted as the first-choice CSF diversion procedure in hydrocephalus secondary to TBM, where technical expertise and experience with this procedure are available.
16	Bhushan et al. [3]	2021	VPS or ETV		The total number of cases with TBMH is 603. The overall success rate in patients who underwent VPS was 51.8%. The overall success rate in patients who underwent ETV – 68%.	The complication rate is more in VPS compared to ETV during the chronic phase of illness.	In the acute phase of illness – VPS is preferred. In the chronic phase of illness – ETV is preferred. Reason – poor anatomy can lead to more complications with ETV in the acute phase of illness.

Outcomes

Results for the patients with TBMH who underwent ETV based on the outcomes of success rate and complications can be found in Table 7.

**Table 7:** The outcome of TBMH patients who underwent ETV. TBMH: tuberculous meningitis hydrocephalus, ETV: endoscopic third ventriculostomy.

Author year publication	Number of patients (n)	Age of the patients	Follow-up period	Good outcome%	Complication%
Husain et al. [10]	n=28	5 months - 68years	3 months to 2.5 years	68%	10%
Singh et al. [11]	n=35	6 months - 32years	12 weeks	77%	
Jha et al. [12]	n=14	9 months - 40years	5 months	64.2%	
Figaji et al. [13]	n=17	<12 years	4–35 months	41.1%	11.7%
Chugh et al. [16]	n=26	7 months - 52 years	1–15 months	73.1%	
Yadav et al. [17]	n=59	6 months - 76 years	7–54 months	58%	22.55%
Goyal et al. [20]	n=24	<18years	6 months	41.7%	16.75%
Aranha et al. [4]	n=26	<18years	5 months	65.4%	3.84%
Bhushan et al. [3]	n=255	1 month - 68 years		68%	3.8% to 22.55%

Interpretation

The average follow-up period in the various studies mentioned above varied from one month to five years. The average outcome success rate of the ETV procedure in the studies mentioned above is 61.8%. However, the complication rate of the ETV procedure varied from 3.84% in the study of Aranha et al. to 16.75% in the study of Goyal et al. [3,4,10,13,17,20]. The complication rate of ETV commonly includes CSF leak, perioperative bleed, blocked stoma, the bulge at the ETV site, and meningitis.

Results for the patients with TBMH who underwent VPS based on the outcomes of success rate and complications can be found in Table 8.

**Table 8:** The outcome of TBMH patients who underwent VPS. TBMH: tuberculous meningitis hydrocephalus, VPS: ventriculoperitoneal shunt.

Author year publication	Number of patients (n)	Age of the patients	Follow-up period	Good outcome	Complications
Lamprecht et al. [9]	n=65	4–131 months	6 months	55.4%	32.3%
Sil and Chatterjee [14]	n=32	1 month to 12 years	4–35 months	25%	43.8%
Srikantha et al. [15]	n=95	1–55 years	3–65 months	Favourable short-term outcome: 33%; favourable long-term outcome: 45%	
Peng et al. [18]	n=19	1 month to 14 years	6–37 months	21.1%	31.57%
Savardekar et al. [19]	n=26	4 months to 11 years	3 months	71.4%	23.5%
Goyal et al. [20]	n=24	<18 years		54.2%	16.7%
Kankane et al. [21]	n=50	3 months to 14 years	3 months	In grade 3–77.5%; in grade 4–30%	10%
Rizvi et al. [22]	n=1038	<18 years	2 weeks to 6 years	48.4% (GOS 5 and 4)	22.11%
Aranha et al. [4]	n=26	<18 years	5 months	65.4%	38.4%
Bhushan et al. [3]	n=348	1 month to 68 years		51.8%	10% to 43.8%

*Interpretation* 

The average follow-up period in the various studies mentioned above varied from two weeks to six years. The average outcome success rate of the VPS procedure in the studies mentioned above is 57.82%. GOS (Glasgow Outcome Scale) and Vellore grading were outcome measures used by a few studies, and some studies used either death or disabilities to determine the outcome. The overall complication rate of the VPS procedure varied from 10% in the study by Kankane et al. to 43.8% in Sil and Chatterjee et al. [3,4,9,14,18-22]. The common complications in VPS patients include shunt infections, shunt obstructions, intraventricular haemorrhage, and multiple shunt revisions.

**Figure 2:**
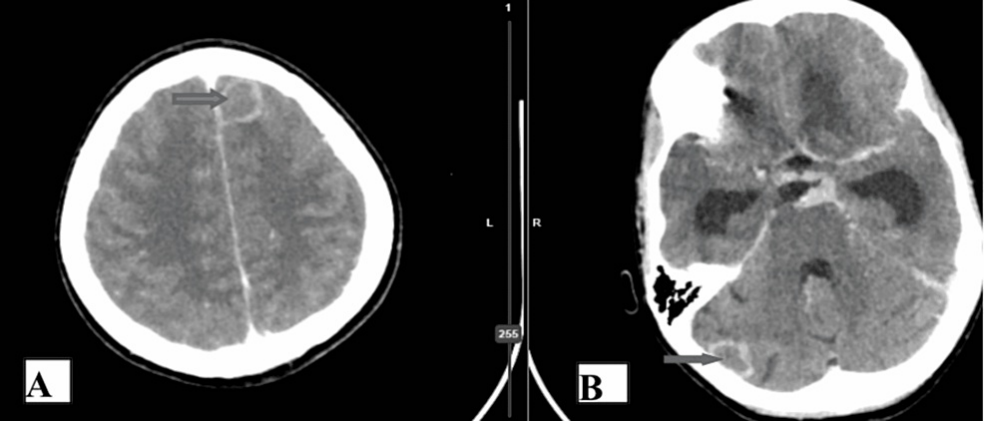
Preoperative CT brain scan (contrast study, axial section) of a patient showing tuberculomas in the left frontal region and right cerebellum.

**Figure 3:**
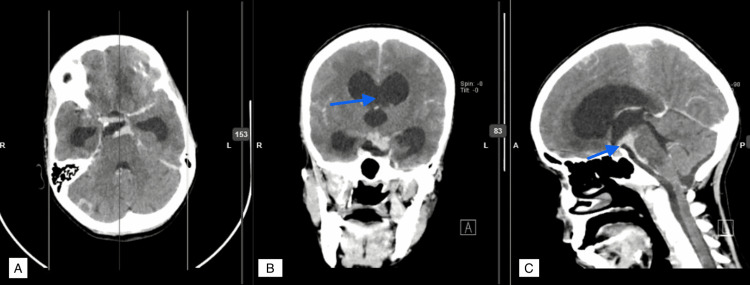
Preoperative CT brain contrast study (A - axial, B - coronal, C - sagittal sections) of the same patient showing dilated bilateral lateral and third ventricle with minimal periventricular seepage and leptomeningeal enhancement.

**Figure 4:**
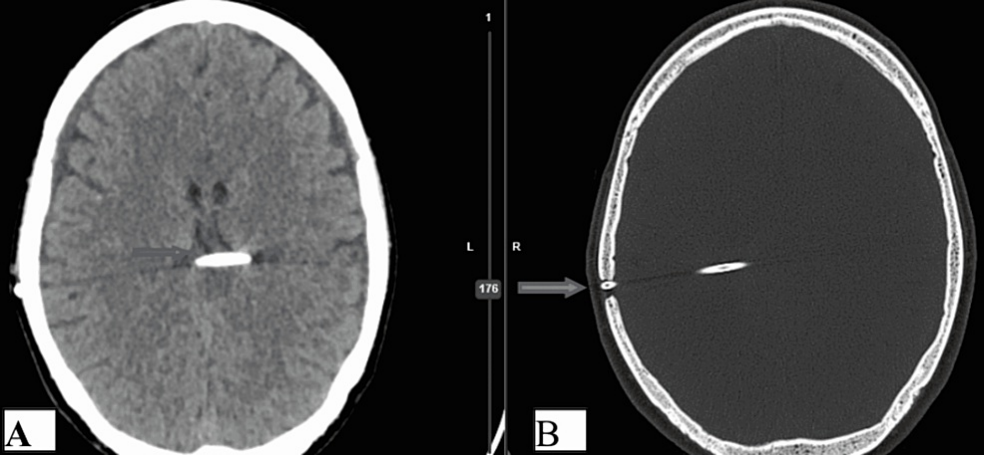
Postoperative CT brain plane study (axial section) and CT bone window of the same patient showing the burr hole defect in right parietal bone, VP shunt in situ with its tip in the trigone of the lateral ventricle.

The preoperative and postoperative CT brain scans of a patient with TBMH who underwent VPS can be found in Figures 2-4.

Discussion

Hydrocephalus is the most frequent complication of TBM and is profoundly more common in children than in adults. Our study comprised 2207 patients with TBMH who underwent either VPS or ETV. Although various studies determined the efficiency of the surgical intervention based on the clinical outcomes and complications, the indications and timing of VPS and ETV were not steady across the studies. In our study pattern, success rates of ETV in patients with TBMH varied widely from 41.1% to 77% [3,4,10-13,16,17,20]. The complication rate in ETV varied from 3.8% in the study of Aranha et al. [4] to 22.55% in Yadav et al. [3,4,10,13,17,20]. The common complications in patients who have undergone ETV include CSF leak, perioperative bleed, blocked stoma, bulge at the ETV site, and meningitis. The presence of advanced clinical-grade, extra CNS TB, dense adhesions in the prepontine cistern, and unidentifiable third ventricle floor anatomy leads to the failure of ETV [12]. Complex hydrocephalus and associated cerebral infarcts are significant causes of failure to improve after ETV [17]. Results of ETV were better in patients without cistern exudates, good nutritional status, and a thin and identifiable floor of the third ventricle. ETV should be better avoided for acute hydrocephalus in patients with tuberculous meningitis and should be reserved for those who have been on ATT for at least four weeks or those in the phase of chronic burnout and hydrocephalus has developed late [1]. Some authors suggested ETV as worth trying before subjecting the patients to VP shunt as it showed good results in both communicating and obstructing hydrocephalus [4,11]. Few studies regarded ETV as the first choice of management in patients with TBMH despite high CSF cell counts, protein levels, and indistinct third ventricular floor anatomy [4]. On the other hand, a few studies suggested ETV as the first management choice and considered VP shunt and EVD in patients with failed ETV based on the clinical-grade [10]. Thus, there has been a lack of uniformity in the indications for performing endoscopic third ventriculostomy (ETV). On the other hand, success rates of VPS in patients with TBMH have varied widely from 21.1% to 77.5% [3,4,9,14,15,18-22]. The complication rate in VPS varied from 10% in the study of Kankane et al. [21] to 43.8% in Sil and Chatterjee [3,4,9,14,18-22]. The common complications in VPS patients include shunt infections, shunt obstructions, intraventricular haemorrhage, multiple shunt revisions, abdominal CSF collections like pseudocyst, subdural hematomas, skin erosions, pneumonia, and meningitis. One of the studies reported that shunt-related complications occurred in four patients and consisted of an obstruction at the lower end of the shunt in three cases, leading to revision. One patient had an infection at the shunt chamber site, leading to skin excoriation and meningitis [20]. A few studies reported that 15.8% of patients expired in the second and fourth postoperative weeks, respectively; among those who had undergone VPS placement, 21.1% of patients had a full recovery without sequelae, and the other 63.2% of patients survived with various sequelae, including paralysis, impaired vision and hearing, mental retardation, and epilepsy [18]. Rizvi et al. suggested that VPS outcome depends upon the clinical severity of TBMH and holds an unpleasant prognosis in HIV-infected patients compared to HIV-uninfected patients [22]. Srikantha et al. suggested a VP shunt as the first choice of management for grade 4 patients with hydrocephalus and recommended it for patients who do not improve with an EVD [15]. A few studies have suggested early VP shunt in patients with non-communicating hydrocephalus [9]. Prognostic factors to rule out the outcome of shunt surgery include the age of the patient, duration of altered sensorium, CSF cell count, and CSF protein levels. However, ETV has the theoretical ascendancy over VPS in enabling the CSF to circulate through the previously inaccessible areas of the brain, which can generally absorb cerebrospinal fluid. ETV also avoids lodging a foreign body in the form of a shunt, hence avoiding complexities like shunt infection, blockage, and abdominal pseudocyst formation [1].

Limitations

The study scale of the ETV group is small compared to the VPS group, and the data extracted from an adult population are inadequate to define any conclusion. In addition, there is a significant shortage of information regarding the follow-up longevity, which might help determine the long-term outcomes and complications of the VPS and ETV procedures and the timing of procedures in patients with TBMH. Finally, apart from the former concerns, there is limited access to the data, and the methods of the studies could be more specific in a better way.

## Conclusions

After much interpretation, it is suggested that clinical grading of the patients is a basic and effective method to determine the management of TBMH. Moreover, after ruling out the significant differences in the average percentages of outcomes and complications followed by VPS and ETV, ETV is suggested in patients with chronic illness because the chances of ETV failure are high during the initial phase. However, the uncertainty of the ETV gradually descends over some time. Therefore, to attain favourable long-term outcomes with ETV in patients with TBM, ETV should be performed after chemotherapy, ATT, and steroids. In addition, ETV can be beneficial over VP shunt because it requires fewer incisions, associated long-term complications are significantly less than VP shunt, and there are no implanted foreign bodies. In contrast, VP shunt is suggested in the acute phase of illness as patients in modified Vellore grading show favourable outcomes compared to ETV.
